# The structure–activity relationship of copper hydride nanoclusters in hydrogenation and reduction reactions†

**DOI:** 10.1039/d3na01145c

**Published:** 2024-02-01

**Authors:** Xi Zheng, Ye Liu, Wanli Ma, Yan Su, Yu Wang

**Affiliations:** a Department of Chemistry, Humboldt-Universität zu Berlin 12489 Berlin Germany wangyuxx@hu-berlin.de; b IRIS Adlershof & The Center for the Science of Materials Berlin, Humboldt-Universität zu Berlin 12489 Berlin Germany; c Key Laboratory of Materials Modification by Laser, Ion and Electron Beams, Ministry of Education, Dalian University of Technology 116024 Dalian China

## Abstract

Copper hydrides are highly active catalysts in hydrogenation reactions and reduction processes. Three Stryker-type copper hydride nanoclusters (NCs), [(TPP)CuH]_6_, [(TCP)CuH]_6_ and [(TOP)CuH]_6_ (TPP = triphenylphosphine, TCP = tricyclohexylphosphine and TOP = tri-*n*-octylphosphine), were synthesized in this study. Due to variations in the electron-donating properties of the phosphine ligands, the UV-visible absorption spectra of the three NCs exhibited notable distinctions. The influence of the phosphine ligands on the effectiveness of the NCs as hydride sources in hydrogenation processes, as well as on the applicability as homogeneous catalysts for reduction reactions, was systematically studied. Due to the highest electron-donating properties of the TOP ligand, [(TOP)CuH]_6_ was found to exhibit superior performance in both hydrogenation reactions and catalytic reduction reactions. Moreover, these hydrophobic NCs worked well as heterogeneous catalysts in the reduction of 4-nitrophenol.

## Abstract 2

Three Stryker-type copper hydride nanoclusters ([CuH]_6_) were synthesized. Variations in the electron-donating properties of phosphine ligands affect the hydrogenation reaction and reduction process activity of [CuH]_6_.

## Graphical abstract

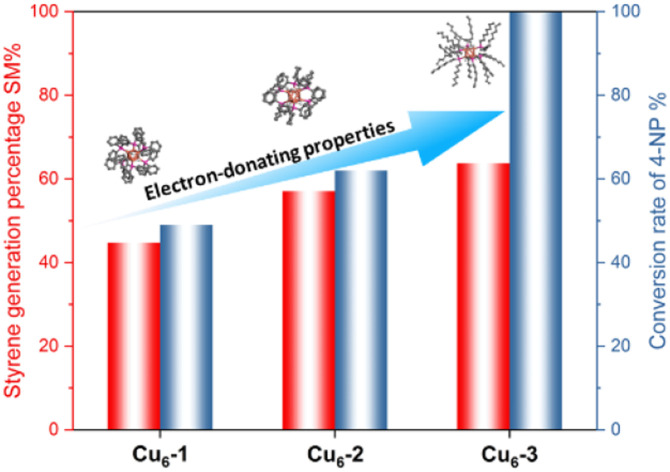

## Introduction

Metal nanoclusters (NCs) have emerged as a noteworthy class of nanomaterials, showcasing distinct properties attributable to the quantum confinement effect when compared to their nanoparticle counterparts.^1–4^ Wet chemistry approaches enable the preparation of metal NCs under ambient pressure on a gram scale.^5,6^ Typically, this synthesis involves the reduction of metal salts or precursors in the presence of protecting ligands.^7^ Due to the strong interaction between metal ions and ligands, strong reducing agents like sodium borohydride are commonly employed.^8^ The structures of ligand-protected metal NCs can be solved by single crystal X-ray diffraction (XRD).^9,10^ Following up on the breakthrough of Au_102_, an increasing number of gold^11–15^ and silver NCs^16–19^ have been isolated and structurally characterized. These NCs exhibit a characteristic structure comprising a metal core shielded by a monolayer of metal–ligand complexes.^20,21^ The overall oxidation state of gold and silver falls between zero and plus one, tending closer to zero.^22^ With a growing number of gold and silver NCs being reported, it is now feasible to explore structure–property relationships at the atomic level.^23^ In the case of copper systems, the scenario is somewhat different. In addition to introduced organic ligands, hydrides are often identified in copper NCs.^24^ Locating these hydride ligands using XRD poses challenges due to the weak diffraction of hydrogen.^25^ The advancement of neutron diffraction has facilitated the identification and location of hydrides in copper NCs.^26^ Consequently, it was discovered that many copper NCs are in fact copper hydride NCs.^27^ The overall oxidation state of copper remains between zero and plus one, albeit leaning closer to plus one.^28,29^

Copper hydrides have become widely used in organic synthesis and catalysis. They are a good source of hydrides in stoichiometric reactions.^30^ Alternatively, catalytic reductions and hydrogenation reactions can be performed in the presence of an additional reducing agent, ensuring the continuous regeneration of copper hydride NCs during the reaction.^31^ One of the most well-known copper hydride NCs is the hexametric [(TPP)CuH]_6_ (TPP = triphenylphosphine), commercially available as Stryker's reagent.^32^ This hexametric NC exhibits notable regioselectivity and chemoselectivity, particularly in the hydrogenation of α,β-unsaturated ketones and aldehydes.^33^ In addition to triphenylphosphine, the hexametric NC can be synthesized using various phosphine ligands. For instance, tri(*p*-tolyl)phosphine and tris(4-methoxy)phenyl phosphine^34^ have been employed to investigate their impact on the electron transfer ability of copper hydride clusters ([CuH]_6_). Despite these efforts, systematic studies exploring the ligand effect on the reactivity of [CuH]_6_, especially considering factors such as steric hindrance^33^ and electronic structures,^34^ are scarce.^35^ The present study utilizes different [CuH]_6_ NCs protected by aliphatic and aromatic phosphines as a model system to comprehensively examine how the ligand influences the catalytic properties of the respective NC. The study reveals that the phosphine ligand with high electron-donating properties plays an important role in both stoichiometric and catalytic reactions. We anticipate that the results presented here will serve as instructive guidance for the future design of NCs with enhanced catalytic properties.

## Experimental

### Chemicals

Copper(ii) acetate anhydrous (Cu(OAc)_2_, 98%) was purchased from Alfa Aesar. Triphenylphosphine (TPP, 99%), tricyclohexylphosphine (TCP, 98%), tri-*n*-octylphosphine (TOP, 97%), 1,1,3,3-tetramethyldisiloxane (TMDSO, 99%), benzene-d_6_ (C_6_D_6_, 99 atom% D), phenylacetylene (98%), sodium borohydride (NaBH_4,_ 98%) and 4-nitrophenol (4-NP, 99%) were purchased from ABCR. *O*,*O*,*O*-1,3,5-trimethylresorcinol (98%) was purchased from Apollo Scientific. Toluene (99.5%), acetonitrile (99.9%), methanol (MeOH, 99.9%), and ethanol (EtOH, 99.9%) were purchased from Carl Roth.

### Synthesis of [CuH]_6_ nanoclusters

All procedures for the synthesis of [CuH]_6_ were performed in an argon-filled glovebox. Cu(OAc)_2_ (0.186 g, 1 mmol), TPP (0.525 g, 2 mmol), and 1,1,3,3-tetramethyldisiloxane (1.2 mmol, 0.22 mL) were stirred in anhydrous toluene (2 mL) for 1 h.^36^ The colour of the reaction mixture changed from blue to green and then to dark red. The solution was filtered and described for [(TPP)CuH]_6_ (Cu_6_-1). The synthesis of [(TCP)CuH]_6_ (Cu_6_-2) and [(TOP)CuH]_6_ (Cu_6_-3) closely mirrored that of Cu_6_-1, with the only difference being the utilization of TCP or TOP in place of TPP, respectively. The hydride of Cu_6_-2 and Cu_6_-3 was obviously found in the ^1^H NMR spectra. More detailed information can be known in the ESI.†

### Characterization methods

UV-vis spectra were recorded on 8453 UV-visible Spectroscopy systems from Agilent with argon purging. ATR-FTIR was recorded inside an Ar-filled glovebox using an Alpha spectrometer (Bruker) with an Alpha-P (Bruker) ATR unit. Nuclear magnetic resonance (NMR) spectra were collected on a Bruker Avance II 300 MHz at room temperature.

### Hydrometallation of alkynes using [CuH]_6_

In the glovebox, phenylacetylene (4.6 × 10^−3^ mmol, 1 equiv.) was mixed with [CuH]_6_ (5.6 × 10^−3^ mmol, 1.2 equiv.) in C_6_D_6_ (520 μL). The solution was stirred vigorously for 1 h and the color of it changed from dark red to light yellow. The vessel was vented to air, and stirring over silica gel precipitated the Cu byproducts. The yield of styrene was determined from the ^1^H NMR spectrum (300 MHz, C_6_D_6_) with an internal stranded (*O*,*O*,*O*-1,3,5-trimethylresorcinol). Methanol (2.47 × 10^−3^ mmol, 0.5 equiv.) and TMDSO (2.7 × 10^−2^ mmol, 5.9 equiv.) were additionally added to the system separately for the control study.

### Heterogeneous catalytic reduction of 4-nitrophenol

A water solution of 4-NP (1 ml, 0.1 mM) with NaBH_4_ (2.5 mg, 6.48 × 10^−2^ mmol) and [CuH]_6_ (1.02 × 10^−3^ mmol) was mixed, and the mixture was stirred for 5 min at room temperature. The intensity of the 4-NP absorption peak centered at 400 nm was monitored as a function of time to follow the reduction of 4-NP.

### Computational methods

In all the calculations, the protecting ligands of all the nanoclusters were simplified as –P(CH_3_)_3_ groups, which had been proved to be an effective way to reduce the computational complexity. The geometric structure was optimized by the Perdew–Burke–Ernzerhof hybrid functional (PBE0) method with Grimme's BJ-damped variant of DFT-D3 empirical dispersion, while the all-electron def2-SVP basis set was used for all the atoms. Time-dependent density functional theory calculations were performed by setting the effective core potential LANL2DZ on all the copper atoms. Orbital composition analysis was performed by the Mulliken method using the Multiwfn 3.8 program.^37^ All the calculations were performed using the ORCA package.^38^

## Results and discussion

**Fig. 1:**
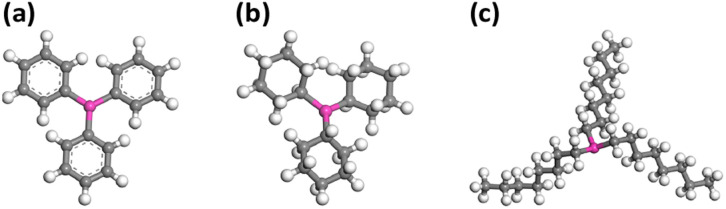
Schematic illustration of the (a) TPP, (b) TCP, and (c) TOP phosphorus ligands of [CuH]_6_. Colour legend: pink P; grey, C; white, H.

It's reported in a Pd-catalysed system that phosphine ligands play a crucial role in determining the reactivity and selectivity.^39^ The stereoselectivity and regioselectivity of the catalytic hydrogenation of ketones, α,β-unsaturated ketones, and aldehydes, facilitated by soluble copper(i) hydride catalysts, have also been extensively investigated by Stryker and co-workers.^31–33^ However, the investigations mainly focused on the impact of extra phosphine ligands introduced additionally into the reaction. It is noteworthy that in their studies, the primary catalyst remains TPP-protected [CuH]_6_. Tolman *et al.* introduced a methodology to clarify the electron donor–acceptor characteristics of phosphine ligands, wherein a lower carbonyl stretching frequency of the ligand indicates a strong electron-donating ability.^40^ Their findings demonstrated that, in contrast to aromatic compounds, aliphatic compounds possess a heightened electron-donating capability. Specifically, linear compounds with aliphatic structures exhibit superior electron-donating properties, which is correlated to the increase of the aliphatic chain length. Hence, the influence of coordinating phosphine ligands on the reactivity of [CuH]_6_ has captivated our attention. Besides TPP, two aliphatic phosphines, namely tricyclohexylphosphine (TCP) and tri-*n*-octylphosphine (TOP), were chosen in this study. Fig. 1 illustrates the structural models of these phosphine ligands.

Cu_6_-1 was synthesized using 1,1,3,3-tetramethyldisiloxane, following the reported procedure in the literature. The synthesis of Cu_6_-2 and Cu_6_-3 mirrored that of Cu_6_-1. ^31^P NMR spectra in C_6_D_6_ indicate that the set of [CuH]_6_ NCs were of high purity with characteristic singlets at 8.66, 24.07, and −6.93 ppm, respectively (Fig. S1†). The variations in the P chemical shifts were ascribed to the different phosphine ligands and their coordination with copper.^41^ The attenuated total reflection-infrared (ATR-IR) experiments on [CuH]_6_ are depicted in Fig. S2.† Taking Cu_6_-1 as an example, the distinct absorption peak at 1435 cm^−1^ is attributed to the characteristic vibration of P–Ar. Additionally, the confirmation of aryl groups in Cu_6_-1 was established through absorptions located at 747 cm^−1^ and 3053 cm^−1^. These absorptions are attributed to the out-of-plane deformations of C–H and stretching vibrations of =C–H, respectively, further confirming the presence of TPP in Cu_6_-1.^42,43^ In Cu_6_-2 and Cu_6_-3 NCs, distinct vibrational bands around 1100 cm^−1^ were attributed to the C–P asymmetric stretching vibration.^44^ The above results suggest that when phosphine ligands are coordinated to copper, the ligand backbone structures underwent no apparent changes.

**Fig. 2:**
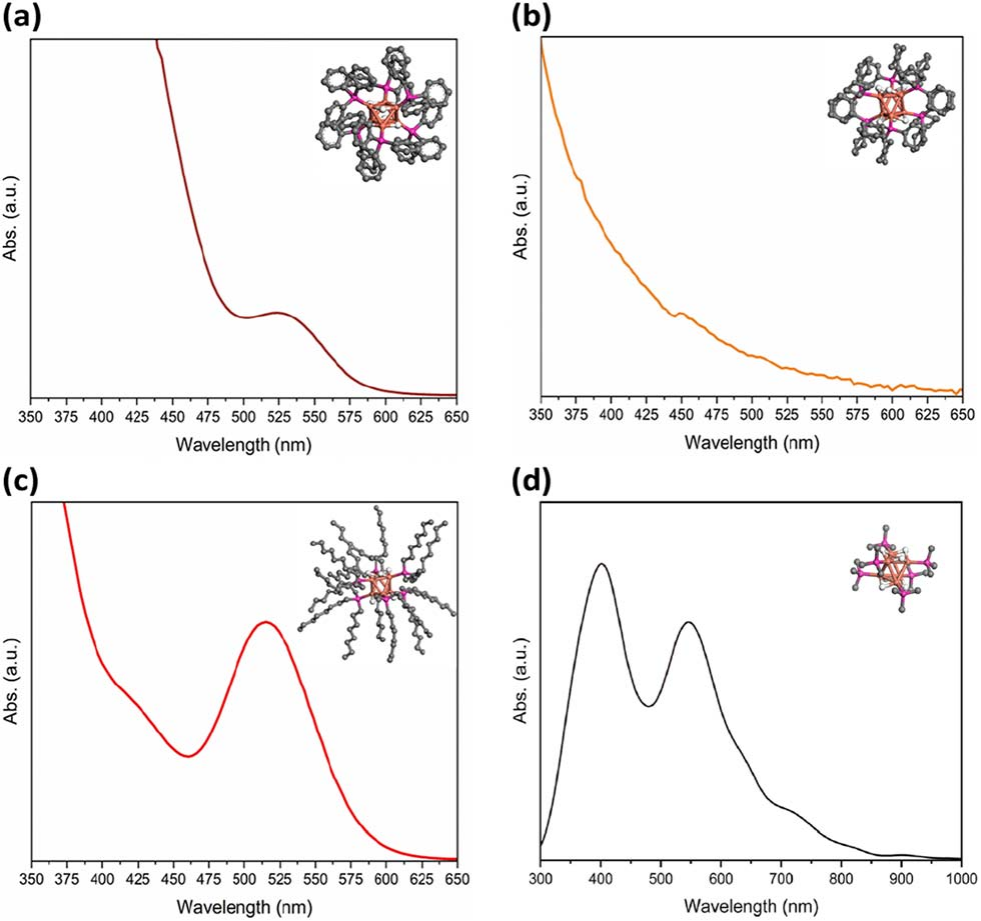
The experimental UV-vis absorption spectra of (a) Cu_6_-1, (b) Cu_6_-2, (c) Cu_6_-3 and (d) TDDFT simulated absorption spectrum of [CuH]_6_ (the Gaussian width was set to 60 nm). Insets show their structures. Colour legend: orange, Cu; pink, P; grey, C; white, H. Hydrogen atoms on ligands are omitted for clarity.

Fig. 2 shows the UV-vis absorption spectra illustrating various absorption characteristics of [CuH]_6_ NCs in toluene. Notably, Cu_6_-1 exhibited an distinctive absorption band centred at approximately 524 nm, consistent with previous findings.^34^ The spectrum of Cu_6_-2 was found featureless. Cu_6_-3 exhibits an absorption peak around 515 nm, shifted slightly compared to Cu_6_-1. The crystal structure of Cu_6_-1 is composed of a octahedron copper core with six long and six short Cu–Cu distances.^45^ The H ligands were assumed to be located on the six long Cu–Cu edges.^46^ Derived from Cu_6_-1, the schemes of Cu_6_-2 and Cu_6_-3 were illustrated.

**Fig. 3:**
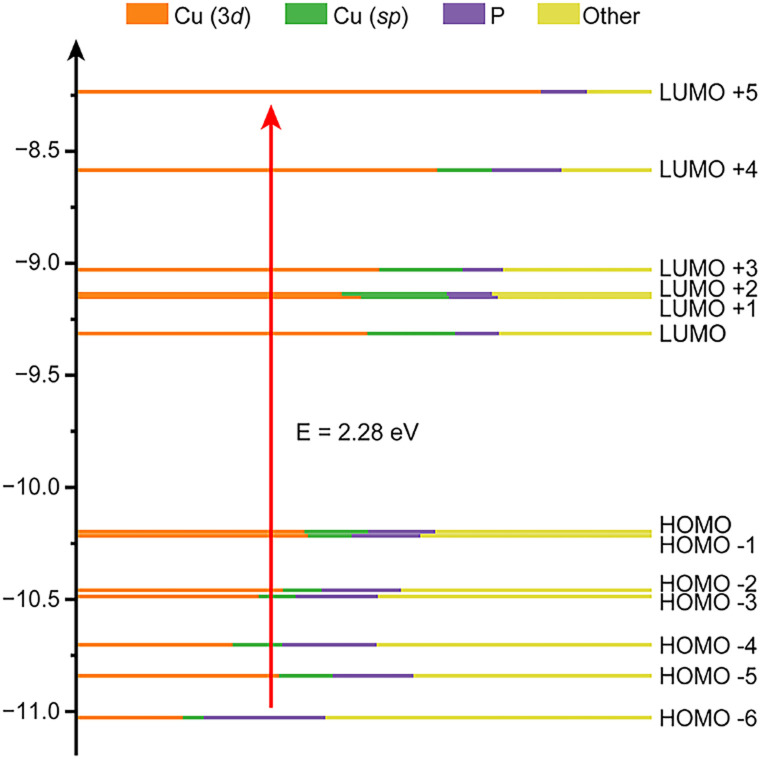
Kohn–Sham (KS) orbitals of the NCs obtained from time-dependent density functional theory (TDDFT) calculations. The different colours in the energy levels show the contribution of the atomic orbitals.

To gain a better understanding of the electronic structure of [CuH]_6_, time-dependent density functional theory (TDDFT) calculations were performed. The theoretical absorption spectrum successfully reproduced one of the experimental peaks at around 2.28 eV (Fig. 2d and S3†). Kohn–Sham (KS) orbital analysis indicates that this absorption predominantly corresponds to the LUMO + 5 ← HOMO − 6 interband transition (Fig. 3, Tables S1 and S2†). Atomic orbital composition analysis suggests that the contributions from P atoms remain significant regardless of the growing contribution of the Cu 3d orbital from HOMO − 6 to LUMO + 5. Since the substituents on the phosphine ligand have great impact on the P atom, their influence on the electronic structure of the NC should also be non-negligible.

**Scheme 1:**
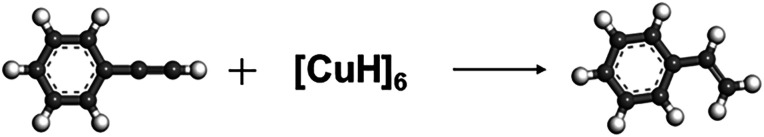
Selective hydrogenation of phenylacetylene by [CuH]_6_.

**Fig. 4:**
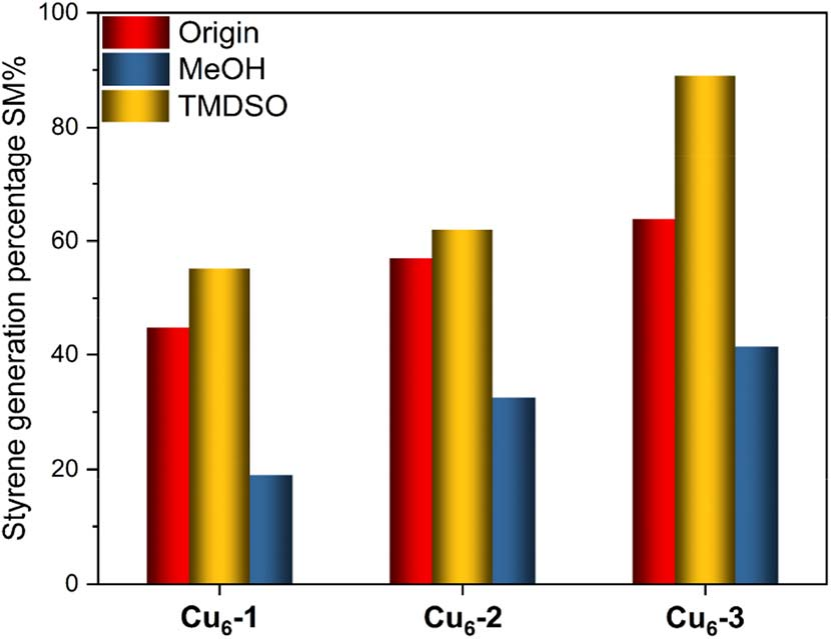
Hydrometallation of phenylacetylene using [CuH]_6_.

To investigate the impact of the ligands on the [CuH]_6_-driven hydrogenation reaction, phenylacetylene hydrogenation was considered as a model reaction (Scheme 1). To our surprise, the three NCs lead to the selective formation of styrene. Although the three NCs showed a similar selectivity, their activities were different. When Cu_6_-3 was used, 63.8% of phenylacetylene was converted to styrene. This value was 44.4% and 57% for Cu_6_-1 and Cu_6_-2, respectively. It is reported that MeOH can react with the hydride in NaBH_4_, forming alkoxyborohydride.^47^ To verify this, we introduced MeOH into the system. As expected, a notable inhibition effect was observed in Fig. 4. The generation of styrene decreased to 18.9%, 32.6%, and 41.3%, respectively. This reduction on activity is possibly due to the consumption of the hydride species associated with Cu by MeOH.^48^ Similar to MeOH, EtOH resulted in a reduction in the generation of styrene (Fig. S4†). This conclusion well agrees with the previous analysis. In addition to serving as a source of hydrides, [CuH]_6_ NCs can also serve as catalysts when other reducing agents are present, ensuring the continual regeneration of [CuH]_6_ during the reaction. When TMDSO as a source of hydrides was introduced, the selective reduction of phenylacetylene was promoted. 55.4%, 62.0%, and 90.0% of styrene were found after 1 hour in Cu6-1, Cu6-2 and Cu6-3 catalysis systems, respectively. In both hydrogenation and catalytic reduction reactions, Cu_6_-3 exhibited superior activity. This could be attributed to the high electron-donating properties of the TOP ligand. The stronger electrostatic force increases the Cu–H bond length, and consequently, enhanced the catalytic activity of copper NCs.^49^ We further investigated the changes of the residue after the hydrometallation reaction. While the ATR-IR spectrum showed no apparent change (Fig. S5†), the ^31^P NMR study, as displayed in Fig. S6,† revealed slight shifts that could be attributed to the altered relative positions of the phosphine ligands and their coordination with copper. Notably, new narrow P peaks at 62.74 ppm and 58.49 ppm were found in Cu_6_-2 and Cu_6_-3 systems, respectively. The formation of these catalytically active species during the hydrogenation process could also be attributed to the weaker interaction between Cu and hydrides in Cu_6_-2 and Cu_6_-3.

**Fig. 5:**
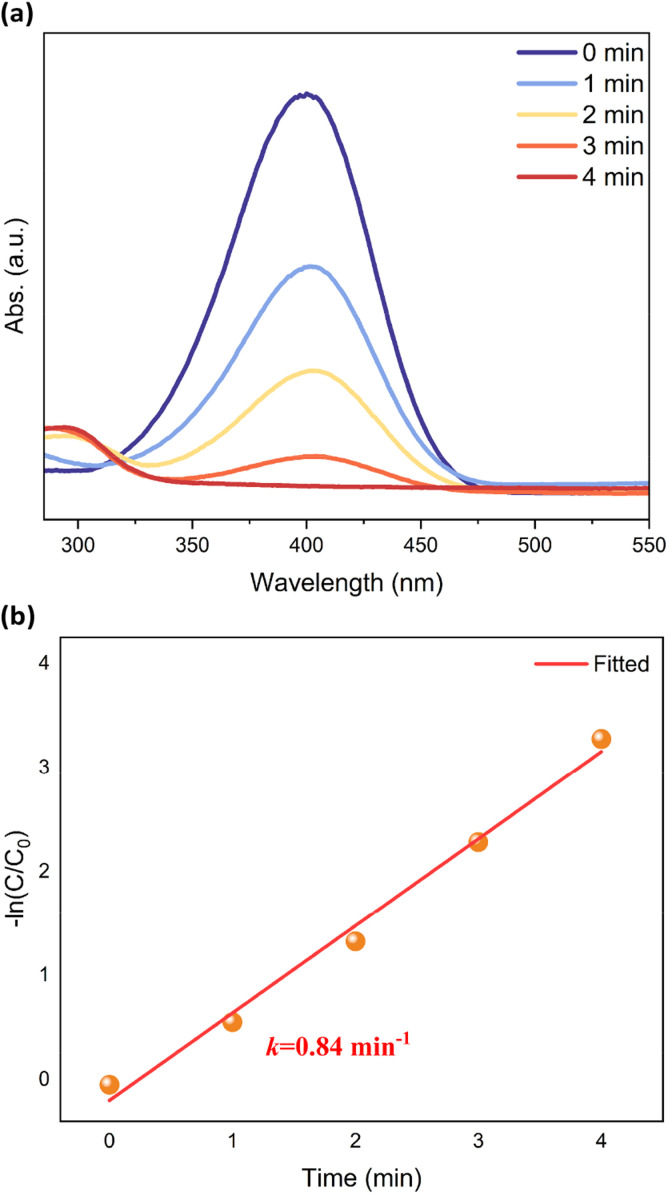
(a) UV-vis spectra showing the gradual reduction of 4-NP in the presence of Cu_6_-3 and (b) plot of −ln(*C*/*C*_0_) *versus* time.

To further investigate the possibility of using [CuH]_6_ as a heterogeneous catalyst, we studied the reduction of 4-nitrophenol (4-NP) to 4-aminophenol (4-AP) by NaBH_4_ as a model reaction. The NCs were dispersed into the solution of 4-NP and NaBH_4_. In the Cu_6_-3 system, 4-NP was completely reduced to 4-AP after 4 minutes. The conversion rate is 49% and 62% for Cu_6_-1 and Cu_6_-2 systems, respectively (Table S3†). Utilizing UV-vis absorption spectroscopy, the reduction process was monitored by determining the intensity variation of the 400 nm peak associated with 4-NP. As the catalytic reaction progressed in the presence of Cu_6_-3, the intensity of the 400 nm peak exhibited a rapid decline, becoming entirely reduced after 4 minutes (Fig. 5a). This observation implies the complete conversion of 4-NP to 4-AP (*λ*_max_ = 295 nm in water). The reaction rate constant was determined to be 0.84 min^−1^ (Fig. 5b), which is much higher than that of other reported coper hydride NCs (0.5 min^−1^ for Cu_11_(Tf-dpf)_6_(OAc)_2_H_3_ and 0.007 min^−1^ for Cu_12_(Tf-dpf)_6_(OAc)_3_ H_3_).^26^ Reusability tests were also carried out on conversion of 4-NP, as shown in Fig. S7 and Table S4.†Cu_6_-3 showed remarkably high stability and recyclability over four cycles of 4-NP reduction. Cu_6_-3 exhibits promise as a catalyst in a variety of applications.

## Conclusions

In summary, three different phosphine ligands were successfully employed in the synthesis of copper hydride NCs, [CuH]_6_. The distinct electron-donating properties of the three ligands resulted in the different reactivities of the three NCs. Due to the highest electron-donating properties of the TOP ligand, [(TOP)CuH]_6_ was found to exhibit superior performance in both stoichiometric hydrogenation and catalytic reduction of phenylacetylene. Moreover, the NCs served as an effective heterogeneous catalyst in the reduction of 4-nitrophenol by NaBH_4_. These findings contribute to a deep understanding of the ligand effect on the performance of copper hydride NCs. We hope it could serve as instructive guidance for the future design of NCs with enhanced catalytic properties.

## Author contributions

Y. W.: conceptualization. X. Z. synthesized all the [CuH]_6_ materials and performed all the physiochemical characterization and hydrogenation experiments. Y. L. and Y. S. conducted DFT calculations. W. M. provided UV-vis tests. Data plotting and analysis were performed by X. Z., W. M., and Y. L. The manuscript was drafted by X. Z. and Y. W. and then revised by all the co-authors.

## Conflicts of interest

There are no conflicts to declare.

## Supplementary Material

NA-006-D3NA01145C-s001
