# Supplementary material for: The structure–activity relationship of copper hydride nanoclusters in hydrogenation and reduction reactions
Source: Nanoscale Adv. 2024 Feb 1;6(5):1374–9. doi: 10.1039/d3na01145c (PMC10898441; doi:10.1039/d3na01145c)
Supplement: NA-006-D3NA01145C-s001 [file NA-006-D3NA01145C-s001.pdf]

Electronic Supplementary Material (ESI) for Nanoscale Advances.
This journal is © The Royal Society of Chemistry 2024

Supporting Information

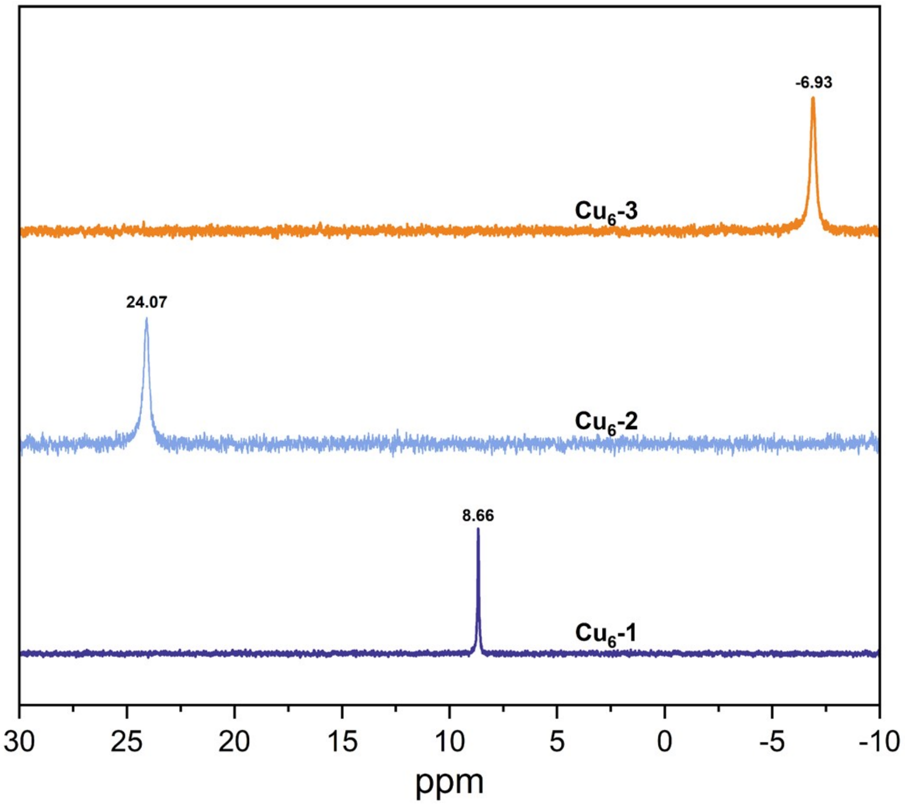

Figure S1. ^{31}P NMR spectrum of $[CuH]_6$ in C_6D_6.

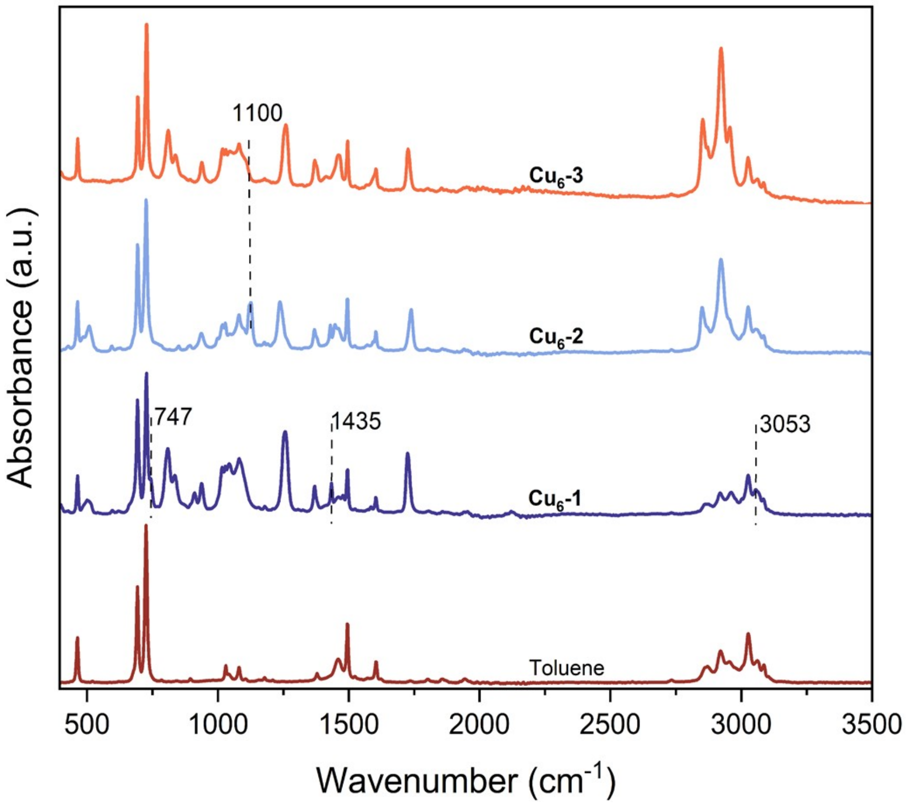

Figure S2. The ATR-IR spectra of the toluene and $[CuH]_6$.

(a)

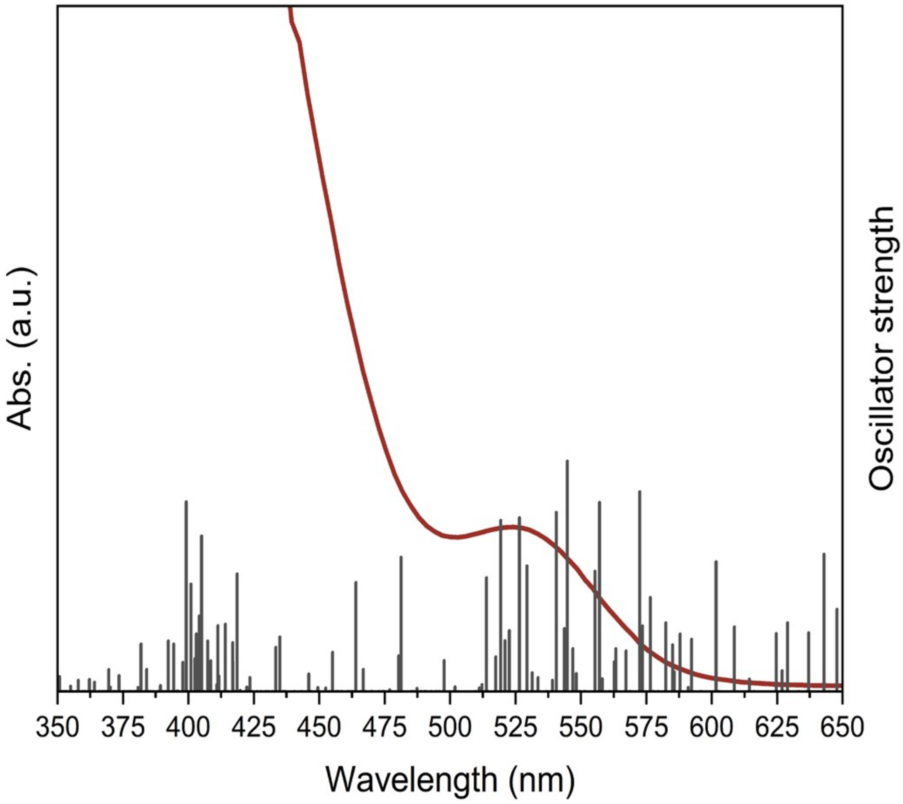
Abs. (a.u.)
Oscillator strength
350
375
400
425
450
475
500
525
550
575
600
625
650
Wavelength (nm)

(b)

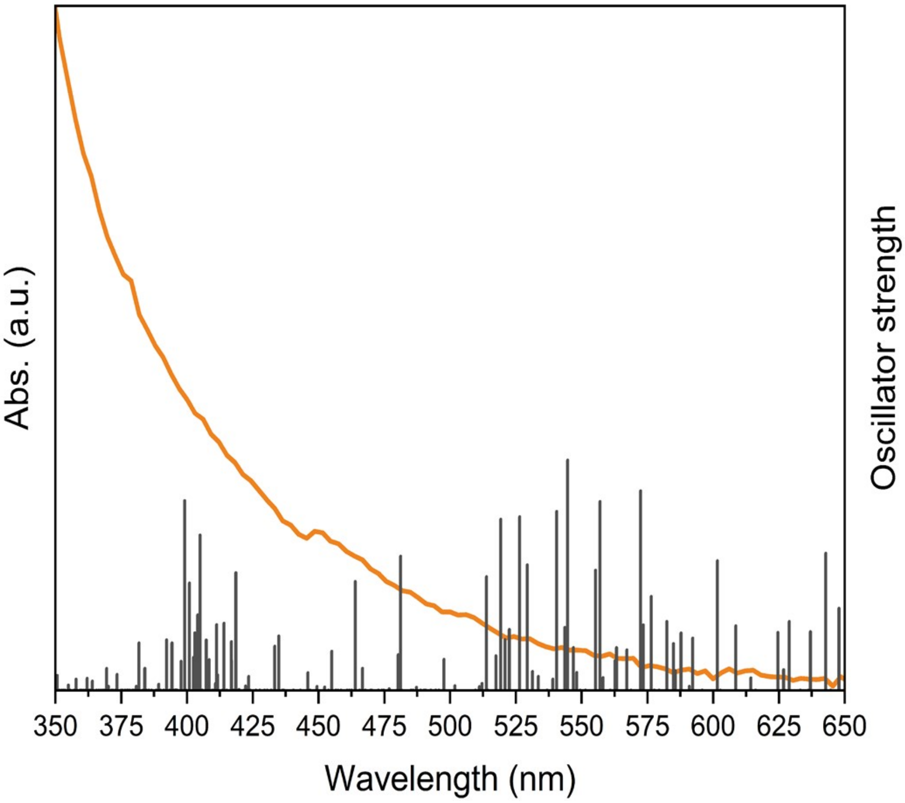
Abs. (a.u.)
Oscillator strength
350
375
400
425
450
475
500
525
550
575
600
625
650
Wavelength (nm)

(c)

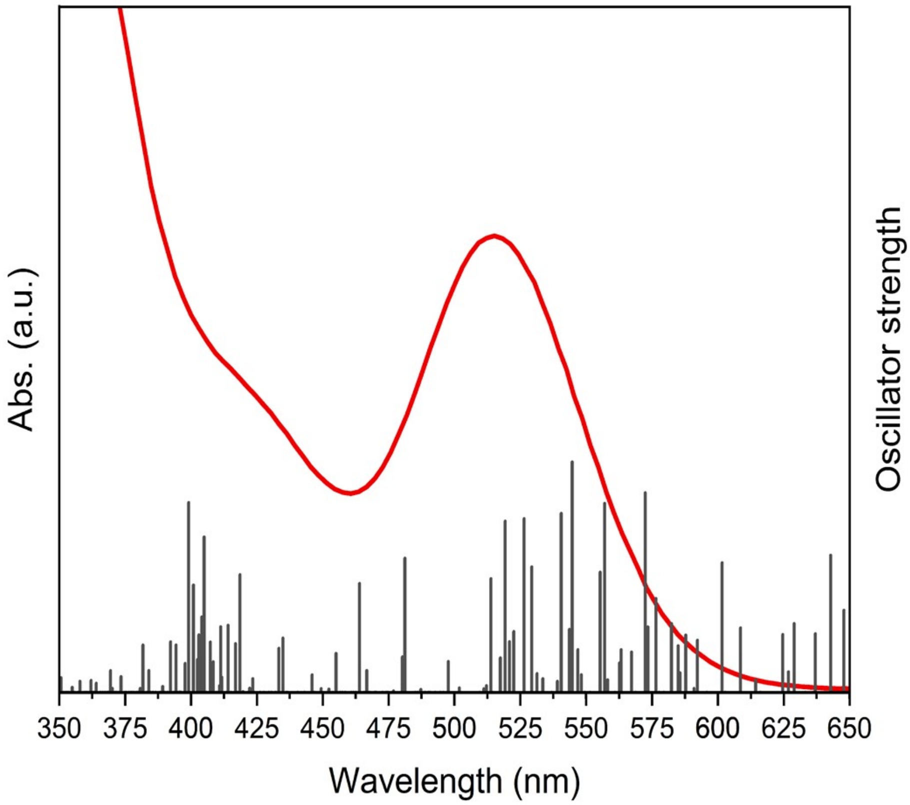

Figure S3. Calculated UV−vis absorption spectra of (a) **Cu_6-1**, (b) **Cu_6-2** and (c) **Cu_6-3**.

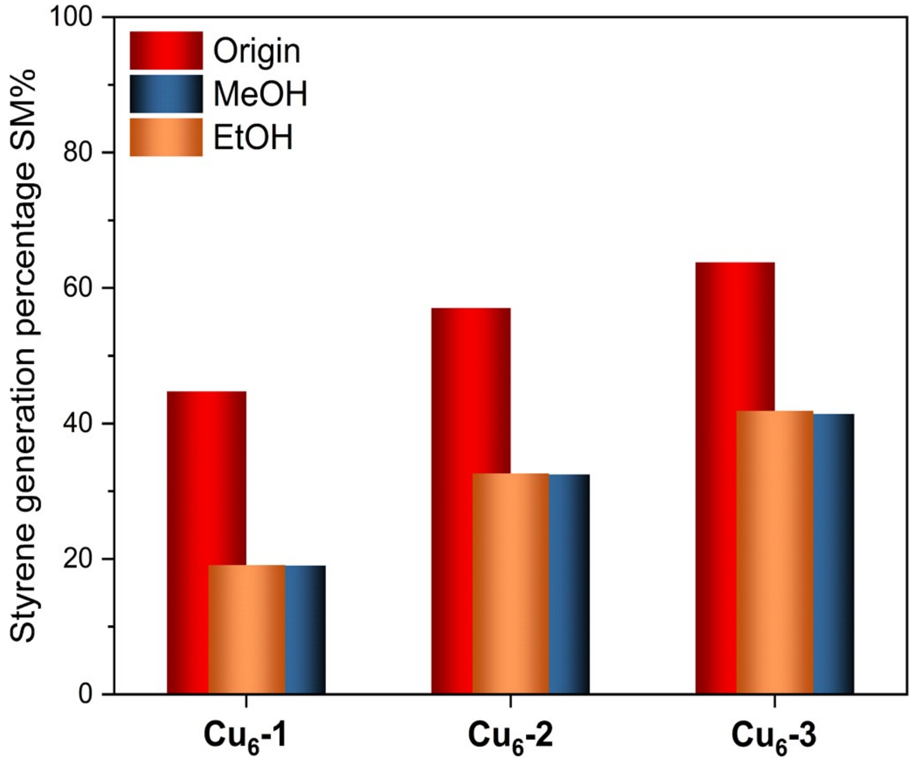

Figure S4. Hydrometallation of phenylacetylene using $[CuH]_6$.

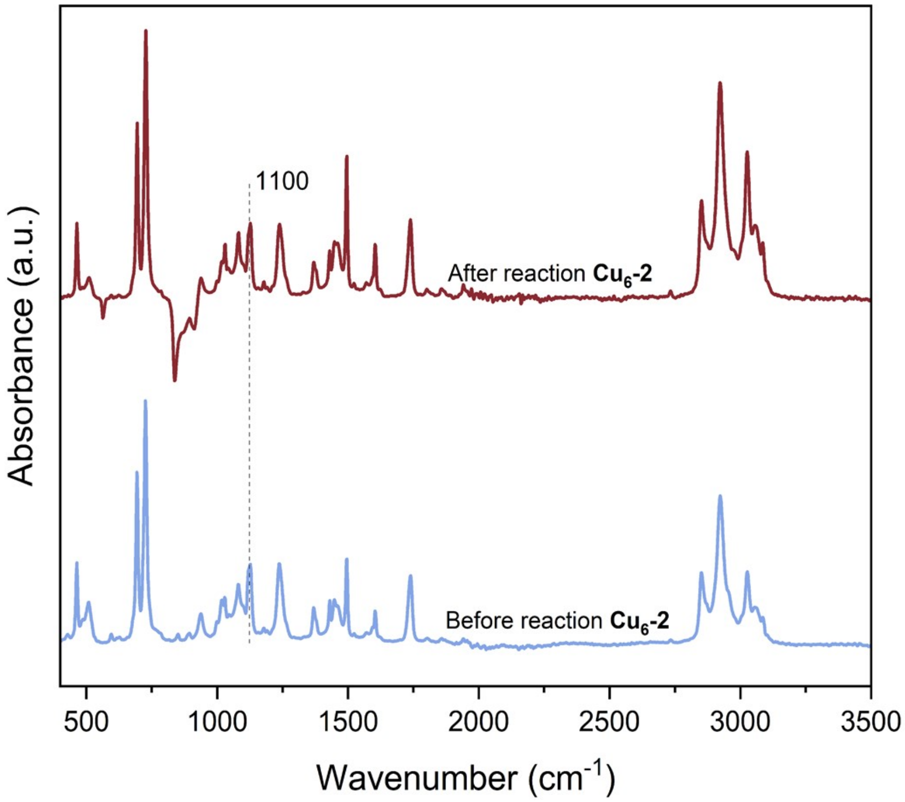

Figure S5. The ATR-IR spectrum of **Cu_6-2** before and after hydrometallation of Alkynes.

(a)

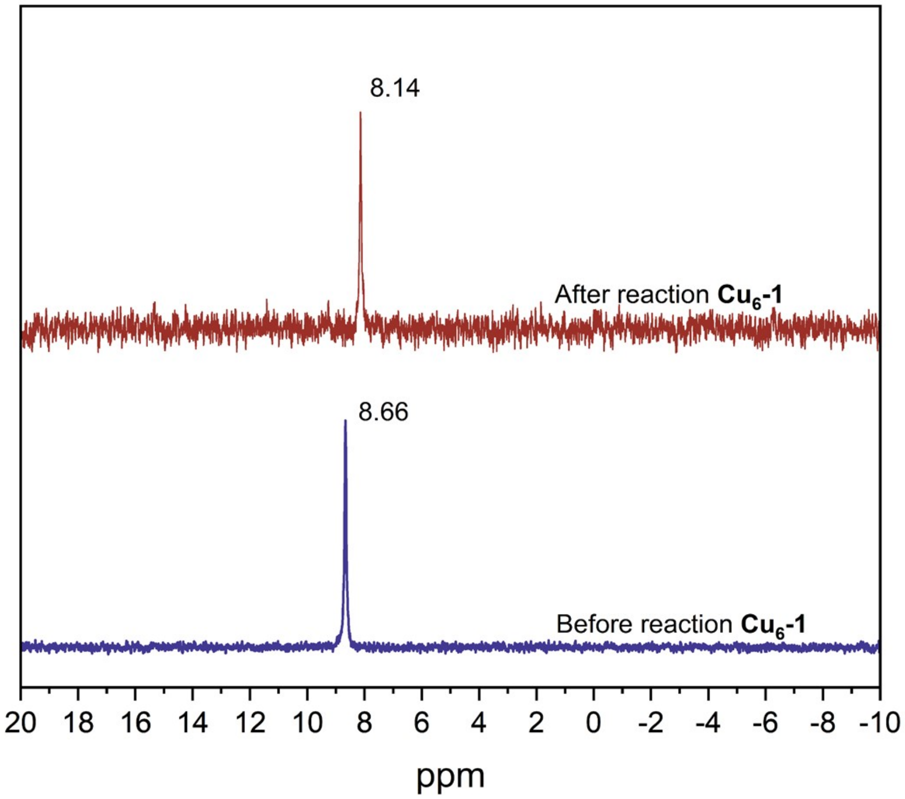
8.14
After reaction Cu_6-1
8.66
Before reaction Cu_6-1
20 18 16 14 12 10 8 6 4 2 0 -2 -4 -6 -8 -10
ppm

(b)

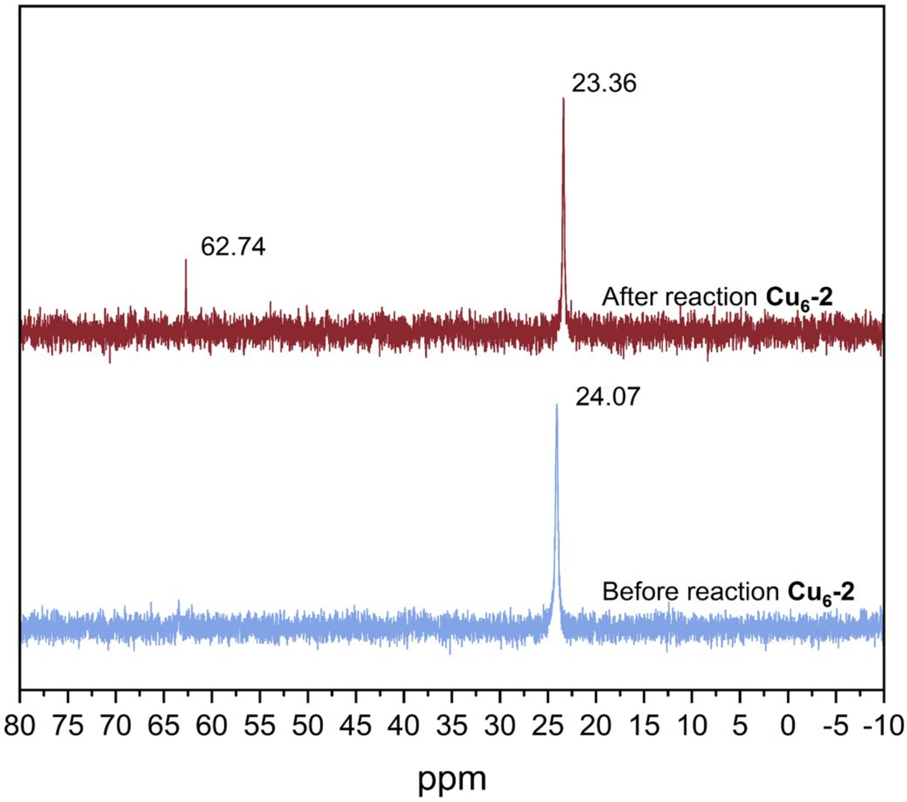
23.36
62.74
After reaction Cu_6-2
24.07
Before reaction Cu_6-2
80 75 70 65 60 55 50 45 40 35 30 25 20 15 10 5 0 -5 -10
ppm

(c)

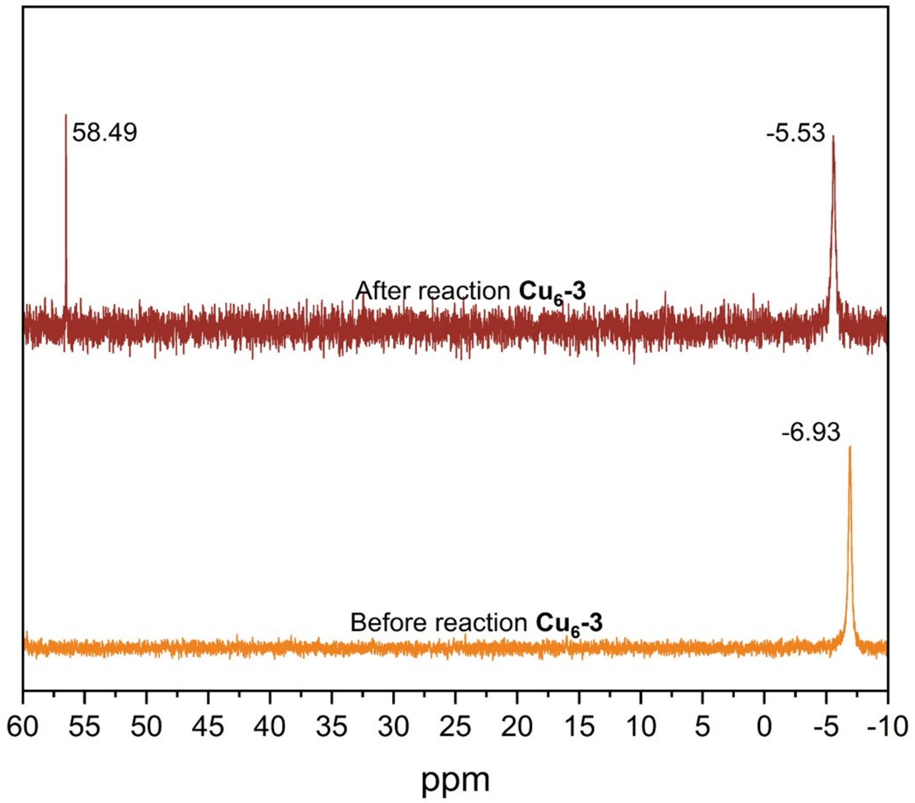

Figure S6. ^{31}P NMR spectrum of (a) **Cu$_6$-1**, (b) **Cu$_6$-2** and (c) **Cu$_6$-3** before and after hydrometallation of Alkynes.

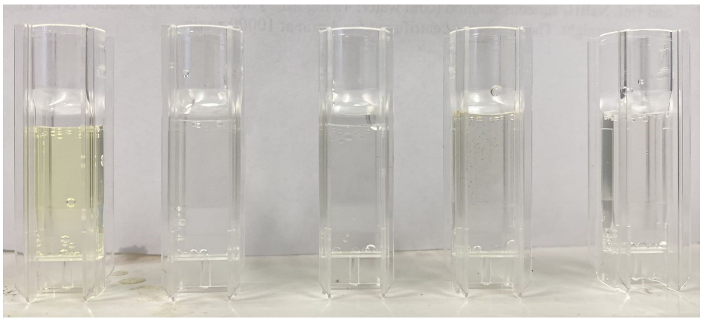

Figure S7. Reusability tests of **Cu_6-3** and changes in solution colour on 4-NP conversion (From right to left indicating the first time to fifth time).

Table S1. Mulliken population analysis of selected HOMO and LUMO energy levels.

Orbital	Energy (eV)	% Cu (3d)	% Cu (sp)	% P
HOMO -6	-11.02	18.28	3.64	21.20
HOMO -5	-10.84	35.06	9.36	14.07
HOMO -4	-10.70	26.99	8.63	16.44
HOMO -3	-10.49	31.51	6.43	14.35
HOMO -2	-10.47	35.75	6.72	13.84
HOMO -1	-10.22	40.11	7.66	11.93
HOMO	-10.21	39.50	11.06	11.70
LUMO	-9.31	50.47	15.28	7.64
LUMO +1	-9.15	49.33	15.31	8.57
LUMO +2	-9.14	46.00	18.30	7.90
LUMO +3	-9.03	52.54	14.49	7.09
LUMO +4	-8.58	62.62	9.55	12.14
LUMO +5	-8.24	80.70	0	8.01

Table S2. The orbital-orbital transitions, energies and oscillator strengths of excited state 130.

Transition	Nature of transitions	Energy (eV)	Oscillator strengths
187 $a \rightarrow$ 216 a	HOMO-29 → HOMO	2.434	0.058
188 $a \rightarrow$ 216 a	HOMO-28 → HOMO	2.263	0.023
188 $a \rightarrow$ 218 a	HOMO-28 → LUMO+1	3.321	0.032
190 $a \rightarrow$ 218 a	HOMO-26 → LUMO+1	3.152	0.020
190 $a \rightarrow$ 219 a	HOMO-26 → LUMO+2	3.157	0.018
192 $a \rightarrow$ 216 a	HOMO-24 → HOMO	2.07	0.027
192 $a \rightarrow$ 217 a	HOMO-24 → LUMO	2.964	0.012
193 $a \rightarrow$ 218 a	HOMO-23 → LUMO+1	3.118	0.026
194 $a \rightarrow$ 218 a	HOMO-22 → LUMO+1	2.948	0.027
195 $a \rightarrow$ 217 a	HOMO-21 → LUMO	2.718	0.037
196 $a \rightarrow$ 219 a	HOMO-20 → LUMO+2	2.869	0.034
199 $a \rightarrow$ 219 a	HOMO-17 → LUMO+2	2.649	0.033
199 $a \rightarrow$ 220 a	HOMO-17 → LUMO+3	2.764	0.047
201 $a \rightarrow$ 220 a	HOMO-15 → LUMO+3	2.674	0.011
203 $a \rightarrow$ 220 a	HOMO-13 → LUMO+3	2.571	0.060
205 $a \rightarrow$ 221 a	HOMO-11 → LUMO+4	2.908	0.022
206 $a \rightarrow$ 222 a	HOMO-10 → LUMO+5	3.16	0.012
208 $a \rightarrow$ 221 a	HOMO-8 → LUMO+4	2.604	0.029
210 $a \rightarrow$ 222 a	HOMO-6 → LUMO+5	2.784	0.170

Table S3. The conversion rate of 4-NP in 4 min

$[CuH]_6$	**Cu_6-1**	**Cu_6-2**	**Cu_6-3**
Conversion rate %	49	62	100

Table S4. Catalysis recycle test of **Cu_6-3**

Number of cycles[a]	Conversion rate %[b]
1	100
2	100
3	100
4	100
5	40

[a]Reaction condition: 4-NP (1 mL, 0.1 mM), $NaBH_4$ (2.5 mg, 6.48 $\times$ 10^{-2} mmol), **Cu_6-3** (1.02 $\times$ 10^{-3} mmol). [b]Monitored by UV-vis absorption spectra.

Spectroscopic Data

Cu_6-2, ^{1}H NMR (500 MHz, C_6D_6) δ 1.20-1.46 (m, 90H), 1.62-1.73 (m, 36H), 1.73-1.81 (m, 36H), 1.91-2.00 (m, 36H), 2.41 (s, 6H)

Cu_6-3, ^{1}H NMR (500 MHz, C_6D_6) δ 0.90-0.92 (m, 54H), 1.36-1.45 (m, 252H), 2.36 (s, 6H)
